# Fabrication of Circular Obelisk-Type Multilayer Microneedles Using Micro-Milling and Spray Deposition

**DOI:** 10.3389/fbioe.2018.00054

**Published:** 2018-05-11

**Authors:** Min Jung Kim, Seok Chan Park, Binod Rizal, Giselle Guanes, Seung-Ki Baek, Jung-Hwan Park, Amy R. Betz, Seong-O Choi

**Affiliations:** ^1^Department of Anatomy and Physiology, Nanotechnology Innovation Center of Kansas State, Kansas State University, Manhattan, KS, United States; ^2^Department of Mechanical and Nuclear Engineering, Kansas State University, Manhattan, KS, United States; ^3^QuadMedicine R&D Centre, QuadMedicine Co., Ltd, Seongnam, South Korea; ^4^Department of BioNano Technology, Gachon BioNano Research Institute, Gachon University, Seongnam, South Korea

**Keywords:** polymer microneedle, transdermal drug delivery, spray deposition, micro-milling, obelisk, circular dichroism, enzyme activity

## Abstract

In this study we present the fabrication of multilayer microneedles with circular obelisk and beveled-circular obelisk geometries, which have potential applications in implantable drug delivery devices. Micro-milling was adopted as an environmental-friendly and cost-effective way to fabricate primary metal microneedle masters. Polylactic acid (PLA) microneedles with sharp tips were then obtained by micromolding followed by oxygen plasma etching and used for preparing polydimethylsiloxane (PDMS) microneedle molds. A spray deposition process was employed for microneedle fabrication to facilitate the formation of multilayer microneedles while helping in maintenance of drug stability. Multilayer microneedles were successfully formed by sequential spraying of poly(lactic-co-glycolic acid) (PLGA) and polyvinylpyrrolidone (PVP) solutions into the mold. The fabricated PLGA-PVP multilayer microneedles penetrated the pig cadaver skin without breakage and released dyes in the skin at different rates, which reveals the potential for implantable microneedles enabling controlled release. Mechanical testing demonstrated that the obelisk-shaped microneedles were mechanically stronger than a pyramid-shaped microneedle and suggested that strong adhesion between PLGA and PVP layers was achieved as well. Structural stability and functionality of a model drug, horseradish peroxidase (HRP), upon spray deposition was examined using circular dichroism (CD) spectroscopy and enzyme activity assay. HRP retained its secondary structure and activity in PVP, whereas HRP in PLGA showed structural changes and reduced activity. Combination of micro-milling and spray deposition would be an attractive way of fabricating drug-containing polymer microneedles with various geometries while reducing prototyping time and process-induced drug instability.

## Introduction

Microneedle-mediated transdermal drug delivery systems have been developed to offer potential benefits over traditional hypodermic injections, such as minimal pain, enhanced patient adherence and self-administration (Kim et al., 2012; Quinn et al., 2014; Prausnitz, 2017). Recently polymer microneedles, particularly dissolving microneedles, have drawn great attention due to their high drug loading capacity, biocompatibility, relatively simple and low-cost fabrication process and reduced medical wastes (Donnelly et al., 2012; Hong et al., 2013; Prausnitz, 2017). Various polymeric materials, including polyvinyl alcohol (PVA), polyvinylpyrrolidone (PVP), polylactic acid (PLA), poly(lactic-co-glycolic acid) (PLGA), polycaprolactone (PCL) and saccharides, have been used for microneedle fabrication (Park et al., 2006; Aoyagi et al., 2007; Chu et al., 2010; Sullivan et al., 2010; Larraneta et al., 2016). Utilizing the dissolution or biodegradation processes of polymers, polymer microneedles have been designed to achieve desirable drug release profiles, typically burst or sustained (Larraneta et al., 2016). Also, previous studies have demonstrated polymer microneedles containing vaccines, small-molecules and peptide, and investigated the efficacy of polymer microneedles through various means, including pharmacokinetics and pharmacodynamics in animal models (Sullivan et al., 2010; Gomaa et al., 2012).

In order to form polymer microneedles, many different fabrication approaches have been explored, such as micromolding (Donnelly et al., 2012), droplet-born air blowing (DAB) (Kim et al., 2013) and *in situ* polymerization (Sullivan et al., 2010), and micromolding is the most widely used method for polymer microneedle fabrication in the literature. Typically micromolding-based fabrication consists of two major processes: (1) master fabrication for replicating molds and (2) casting of solutions containing polymer and active pharmaceutical substances into the mold. So far, various geometries of microneedle masters have been mostly fabricated by well-established microfabrication technologies, such as photolithography and dry/wet etching processes (Henry et al., 1998; Kim et al., 2012; Indermun et al., 2014). Although those techniques can produce complicated structures with high accuracy, fabrication process requires a clean manufacturing environment and highly specialized equipment. For micromolding, a drug-polymer mixture is usually prepared at high concentrations to reduce processing time and cast into the microneedle mold by applying external forces, such as centrifugation and vacuum suction (Perennes et al., 2006; Lee et al., 2008; Hirobe et al., 2015; Mistilis et al., 2015; Rouphael et al., 2017). For example, Lee et al. fabricated dissolving microneedles by casting carboxymethyl cellulose (CMC) into a mold using the centrifugal force (Lee et al., 2008). They prepared highly viscous 27% CMC hydrogel and centrifuged hydrogel-loaded molds at 3,000 × g for up to 2 h to form microneedles. In addition to highly concentrated solutions and excessive external pressure, micromolding occasionally requires high temperatures, which could be a major obstacle to incorporate temperature-sensitive drug molecules in microneedles (Miyano et al., 2005; Park et al., 2005, 2006). Current casting methods, therefore, could be incompatible with labile substances (e.g., peptide and protein drugs). Recently DAB technique was shown to possess great advantages in fast microneedle manufacturing under ambient processing conditions (Kim et al., 2013). In the DAB process, microneedles are formed by stretching polymer droplets followed by air blowing. Since the DAB process does not require master structures and long drying time, microneedle fabrication is very fast and cost-effective, which would be suitable for mass production and commercialization. However, the resulting needle geometry is not diverse so that the DAB process would have limitations in controlling penetration depth, drug loading capacity and mechanical behavior.

**Figure 1:**
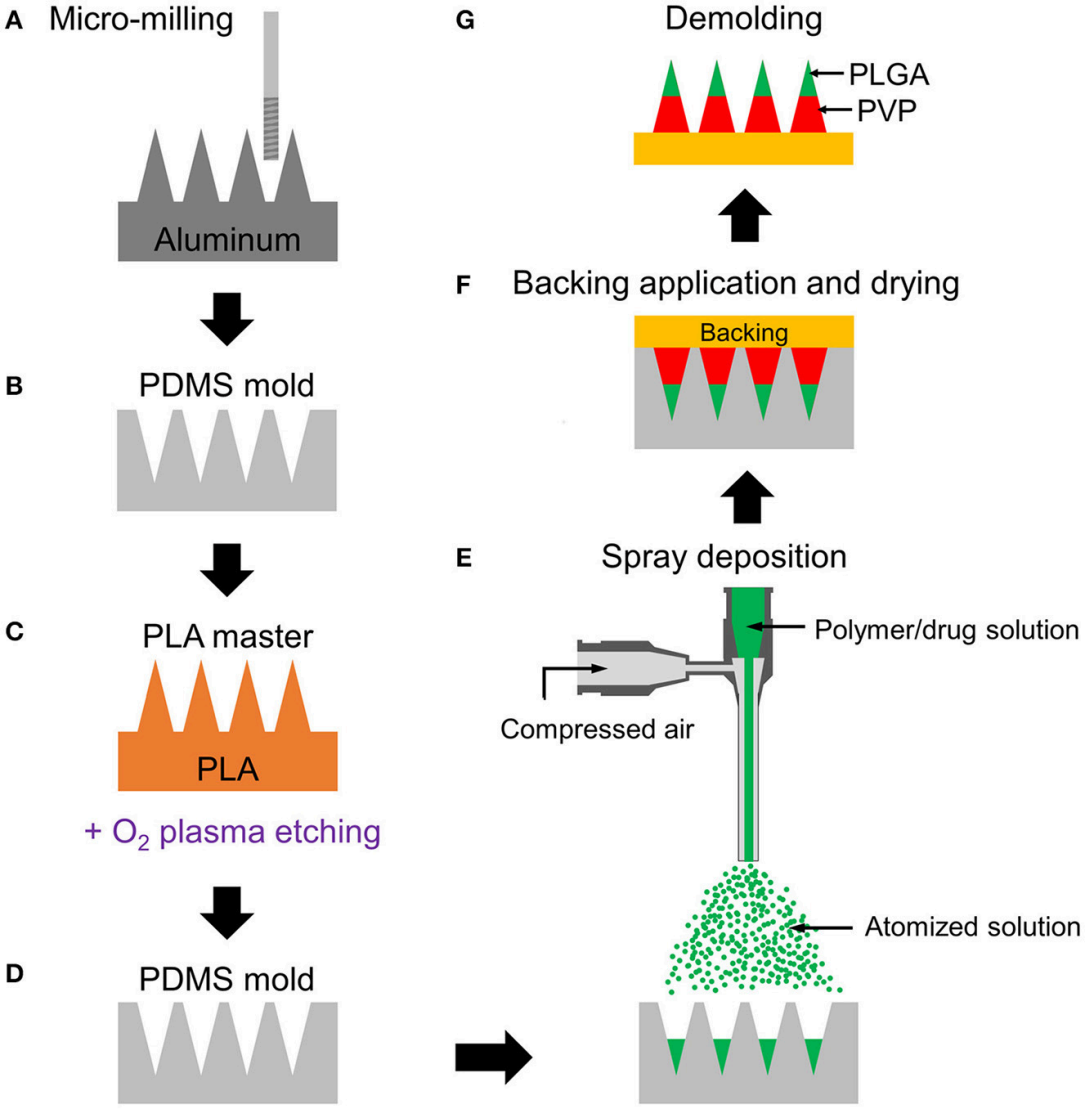
Schematic fabrication process of multilayer microneedles. **(A)** Aluminum master fabrication using micro-milling. **(B)** Replication of PDMS mold from the master. **(C)** Fabrication of PLA master by micromolding and tip-sharpening using oxygen plasma. **(D)** Replication of PDMS mold from the PLA master. **(E)** Spray deposition of drug-containing polymer solution to fill the mold cavity. Multilayer microneedle is formed by sequential deposition of polymer solutions. **(F)** Application of backing material (yellow) on the mold and drying at room temperature for polymer solidification. **(G)** Demolding solidified multilayer microneedle array from the mold. Green and red represent PLGA and PVP layers, respectively.

Recognizing the limitations of current fabrication approaches, we demonstrate the fabrication of obelisk-shaped multilayer microneedles, which could achieve reliable skin penetration, reduce drug degradation during fabrication and have the potential to be used as implantable drug delivery devices with controlled release capability, by combining micro-milling and spray deposition techniques. Previous studies have demonstrated that micro-milling is capable of producing various microneedle geometries with a reproducible and environmental-friendly manner (Bediz et al., 2014) and pyramid-shaped polymer microneedles can be formed by spray deposition (McGrath et al., 2014; Kim et al., 2017). In this study, the spray deposition process was firstly employed to construct obelisk-shaped multilayer microneedles by sequential deposition of PLGA and PVP solutions into microneedle molds, which were replicated from obelisk-shaped master structures (circular-obelisk and beveled-circular obelisk). Circular obelisk-type geometries having a truncated cone base were chosen for facilitating the spray deposition process and for improving skin penetration capability (Bediz et al., 2014). The detailed fabrication process is described in Figure 1. Mechanical characteristics of microneedles were studied by compression tests and *ex vivo* porcine skin insertion tests. Furthermore, the influence of the spray deposition process on drug stability was investigated using horseradish peroxidase (HRP).

## Materials and methods

### Materials

Polyvinylpyrrolidone (PVP, MW 10, and 40 kDa), sulforhodamine B (SRB) and coumarin 314 fluorescence dyes were purchased from Sigma-Aldrich (St. Louis, MO, USA). Poly(DL-lactide-co-glycolide) (50:50 PLGA, ester-terminated, inherent viscosity 0.26–0.54 dL/g) was purchased from Lactel Absorbable Polymers (Birmingham, AL, USA). Poly(lactic acid) (PLA, Ingeo 4043D, 98% L-lactide, with weight average molecular weight of 111 kg/mol) was purchased from NatureWorks (Minnetonka, MN, USA). HRP (lyophilized powder, 85 unit/mg), ethyl acetate, dimethyl carbonate, ethanol, glycerol (all certified ACS grade) and tissue marking dye (Richard-Allen Scientific^TM^ Mark-It^TM^, blue) were purchased from Fisher Scientific (Waltham, MA, USA). Polydimethylsiloxane (PDMS, Sylgard 184) was purchased from Dow Corning (Midland, MI, USA). Multipurpose aluminum blocks (alloy 6061) were purchased from McMaster-Carr (Elmhurst, IL, USA). End mills were purchased from Ultra-Tool (Huntington Beach, CA, USA).

### Aluminum primary master fabrication using micro-milling

**Figure 2:**
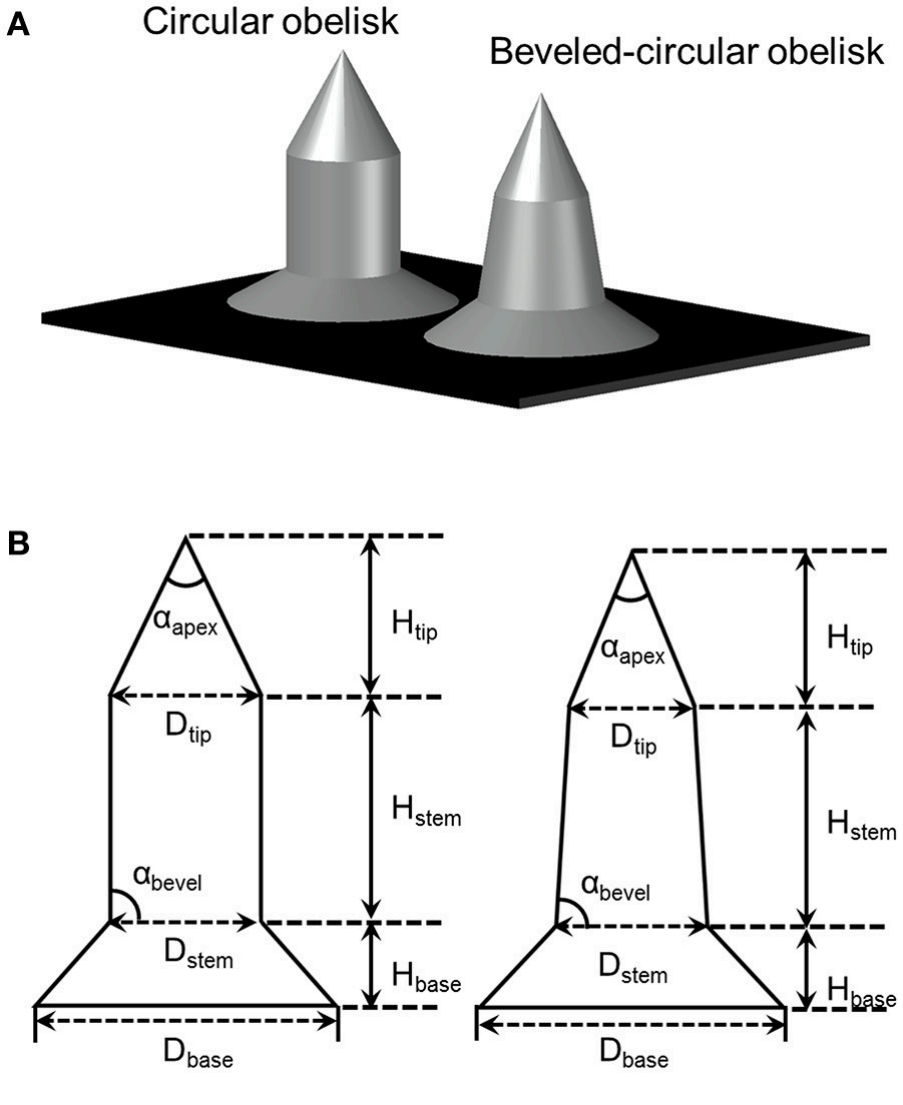
**(A)** Illustration of obelisk-type microneedles. **(B)** Dimension parameters of circular (left) and beveled-circular (right) obelisks.

Two different geometries of microneedles, circular and beveled-circular obelisks, were fabricated by micro-milling of 6061 aluminum alloy. The design of microneedles is depicted in Figure 2A. A blank aluminum work piece (1″ × 1″ × 0.25″, W × L × T) was secured in a computer controlled miniature milling system with the motion accuracy of 1 μm (Minitech Machinery Corporation, Norcross, GA, USA). G-code was written for the fabrication of each structure, and the milling system was controlled by Mach3 CNC controller software. Solid carbide end mill (1/8″ diameter) and aluminum titanium nitride (AlTiN)-coated solid carbide micro end mill (0.01″ diameter) were used for machining a microneedle base and microneedles, respectively. Cutting feed rate and axial depth of cut (per pass) for the circular obelisk microneedles were 4 mm/min and 5 μm, respectively. The beveled-circular obelisk shape was machined with the cutting feed rate of 8 mm/min and axial depth of cut of 7.5 μm. The spindle speed of 20,000 rpm was used for both processes.

### PLA secondary master preparation

From the aluminum primary masters, polymeric secondary masters were replicated by casting PLA into a micromold prepared by PDMS micromolding. A PDMS prepolymer solution (base:curing agent 10:1 w/w) was degassed, poured onto the primary master and cured for 1 h at 90°C. After forming PDMS molds, PLA pellets were stacked in the mold and melted in a vacuum oven (MTI Corporation, Richmond, CA, USA) under −70 kPa for 2 h at 180°C. Vacuum was applied to remove the bubbles entrapped in molten PLA and facilitate the molding process. The replicated PLA structures were separated from the mold after cooling down to room temperature and were subsequently subjected to oxygen plasma etching (COVANCE Plasma System, Femto Science Inc., Yongin, Republic of Korea) at 500 mTorr, 50 Hz, 100 W with 40 sccm O_2_ flow rate for 2 h for sharpening microneedle tips. The plasma treated PLA structures were used for preparing microneedle molds used throughout this study. Microneedle molds were prepared by casting a mixture of PDMS base and curing agent (10:1 w/w) onto the PLA master and cured at 50°C for 3 h.

### Microneedle fabrication using spray deposition process

A commercial coaxial needle (Ramé-hart instrument co., Succasunna, NJ, USA) was used as an atomizing spray nozzle. The outer needle (15 gauge) was connected to a compressed air source, and a solution was fed through the inner needle (21 gauge) by a syringe pump (AL-1000, World Precision Instrument, Sarasota, FL, USA). To fabricate PVP and PLGA microneedles using the spray deposition process, polymer solutions were prepared and sprayed with specific spraying parameters (Table 1) and dried for 15 min at room temperature. The same procedure was repeated five times to form microneedles. A backing layer composed of 40% w/v PVP (MW 40 kDa) and 2.5% v/v glycerol in deionized water was then applied to all the fabricated microneedles and dried overnight at room temperature.

**Table 1:** Summary of solution compositions and spraying parameters.

**Polymer**	**Solution composition**		**Spraying parameters**			
	**Conc. (w/v %)**	**Solvent**	**Flow rate (ml/min)**	**Pressure (psi)**	**Distance (cm)**	**Spraying duration (s)**
PLGA	1.0	A mixture of ethyl acetate and dimethyl carbonate (1:4 v/v)	1.00	5	5	3
PVP	5.0	DI water	0.25	25	5	5

Similarly, PLGA-PVP multilayer microneedles were fabricated by sequential deposition of PLGA and PVP solutions using the spraying parameters described in Table 1. PLGA solution was firstly sprayed onto the microneedle mold, dried for 15 min, and PVP solution was sprayed. To distinguish each layer, SRB and coumarin 314 were added to the aqueous PVP and organic PLGA solutions, respectively. The fabricated multilayer microneedles were visualized using an inverted fluorescence microscope (Olympus IX-73, Tokyo, Japan) coupled with a digital camera and integrated software.

### Mechanical testing of microneedles

The mechanical behavior of spray-formed microneedles was examined by a universal static testing system (5943 single column materials testing system, Instron, Norwood, MA, USA). The fabricated microneedles were affixed to the lower compression platen using double-sided tape and pressed by the upper platen with a test speed of 1.3 mm/min according to ASTM D695-15 (ASTM Standard D695-15, 2015). Force-displacement curves were generated until the displacement reached 500 μm. Single-layer microneedles were formed from either PVP or PLGA to examine the effect of materials on mechanical characteristics. For PVP microneedles, square pyramidal microneedles (300 μm base width, 600 μm height, center-to-center spacing 600 μm; Kim et al., 2017) were also tested, in addition to obelisk-type structures, to examine the effect of geometry on mechanical stiffness. All PVP microneedles were fabricated using the same solution compositions and spray parameters. Finally, PLGA-PVP multilayer microneedles were tested to identify potential mechanical failure at the interface between PLGA and PVP. For each microneedle geometry, five 3 × 3 arrays were subjected to the test. The obtained data were averaged and represented as force per needle vs. displacement with standard deviation.

### Microneedle insertion study

Skin penetration capability of the spray-formed microneedles was examined *ex vivo* using porcine skin samples obtained from the necropsy facilities at Kansas State University in accordance with the Guidelines for Care and Use of Laboratory Animals of Kansas State University. After removing fat and hair using a razor, microneedles were pressed onto the skin with thumb pressure and then removed after 5 min. Total five microneedle patches for each geometry were used for the insertion study. Microneedle insertion sites on the skin surface were selectively stained using a blue tissue-marking dye (Richard-Allen Scientific^TM^ Mark-It^TM^, blue). For histologic examination, the skin samples were embedded in optimal cutting temperature (OCT) compound, snap-frozen in 2-Methylbutane anhydrous, cryosectioned into 10 μm-thick slices using a crystat microtome (Leica CM3050S, Wetzlar, Germany) and examined under a microscope (Olympus IX-73, Tokyo, Japan).

### Enzyme integrity and activity measurement

The influence of the spray deposition process on the enzyme stability and activity was examined using HRP as a model enzyme. HRP-containing PLGA and PVP matrices were fabricated using the spraying parameters described in Table 1. 5.0% (w/v) PVP solution containing HRP (final concentration 0.6 mg/ml) was prepared in 0.1 M potassium phosphate buffer (pH 6). To prepare PLGA solution containing emulsified HRP, 1 ml of HRP solution (2.0 mg/ml) containing an emulsifier (2% w/v PVA) in deionized (DI) water was dispersed in 15 ml of a mixture of ethyl acetate and dimethyl carbonate (1:4 v/v ratio) containing 1.0% w/v PLGA. This two-phase system was emulsified for 1 min at 50% amplitude with a probe sonicator (VCX 130, Sonics & Materials, Inc., Newtown, CT, USA). The prepared PVP and PLGA solutions containing HRP were sprayed on a circular PDMS substrate with a diameter of 10 cm, which mimics the surface of PDMS microneedle mold. HRP in the PVP matrix was obtained by dissolving the sample in 1 ml of 0.1 M phosphate buffer (pH 6). To extract HRP from the PLGA matrix, the sample was dissolved in 5 ml of acetone for 10 min, and the precipitated HRP was collected after centrifugation at 25,800 × g for 15 min. The collected HRP pellet was re-dissolved in 0.1 M phosphate buffer (pH 6), and the amount of HRP was quantified using the Bradford protein assay kit (Pierce^®^ Coomassie Plus Assay Kit, ThermoFisher, Waltham, MA, USA) according to the manufacturer's instructions.

The secondary structure of HRP was examined by circular dichroism (CD) using a Chirascan spectropolarimeter (Applied Photophysics, Leatherhead, United Kingdom). CD in the UV range (200–250 nm) was monitored with a 0.1 mm pathlength quartz cell with HRP concentration of 200 μg/ml. CD data were expressed in terms of molar residue ellipticity, [⊖], in deg cm^2^ dmol^−1^. The fraction of different secondary structures was estimated from the obtained CD spectra by the Dichroweb software package (Lobley et al., 2002; Whitmore and Wallace, 2004, 2008) with the CDSSTR algorithm (Sreerama et al., 2000).

The enzymatic activity of HRP extracted from the polymer matrices was determined by enzymatic conversion of a chromogenic substrate 3,3′,5,5′-tetramethylbenzidine (TMB). The assays were performed in 96-well microplate as follows: 100 μl of HRP samples extracted from PVP and PLGA matrices were mixed with 50 μl of TMB (0.4 g/L) and 50 μl of H_2_O_2_ solution (0.02%) in a citric acid buffer solution followed by shaking of the plate for 30 s at 500 rpm to ensure good mixing. After incubating for 10 min at room temperature, the enzymatic reaction was stopped by adding 100 μl of 1 M phosphoric acid into the microplate wells, and the absorbance at 450 nm was immediately measured for each well. For comparison, native HRP and acid-treated (0.1 M hydrochloric acid) HRP were used as controls.

The assay was performed three times independently, and each sample was measured in triplicate. Data were compared using a Student's *t*-test with equal variances. All data were presented as mean ± standard deviation. A value of *p* < 0.05 was considered statistically significant.

## Results

### Fabrication of microneedle masters

**Figure 3:**
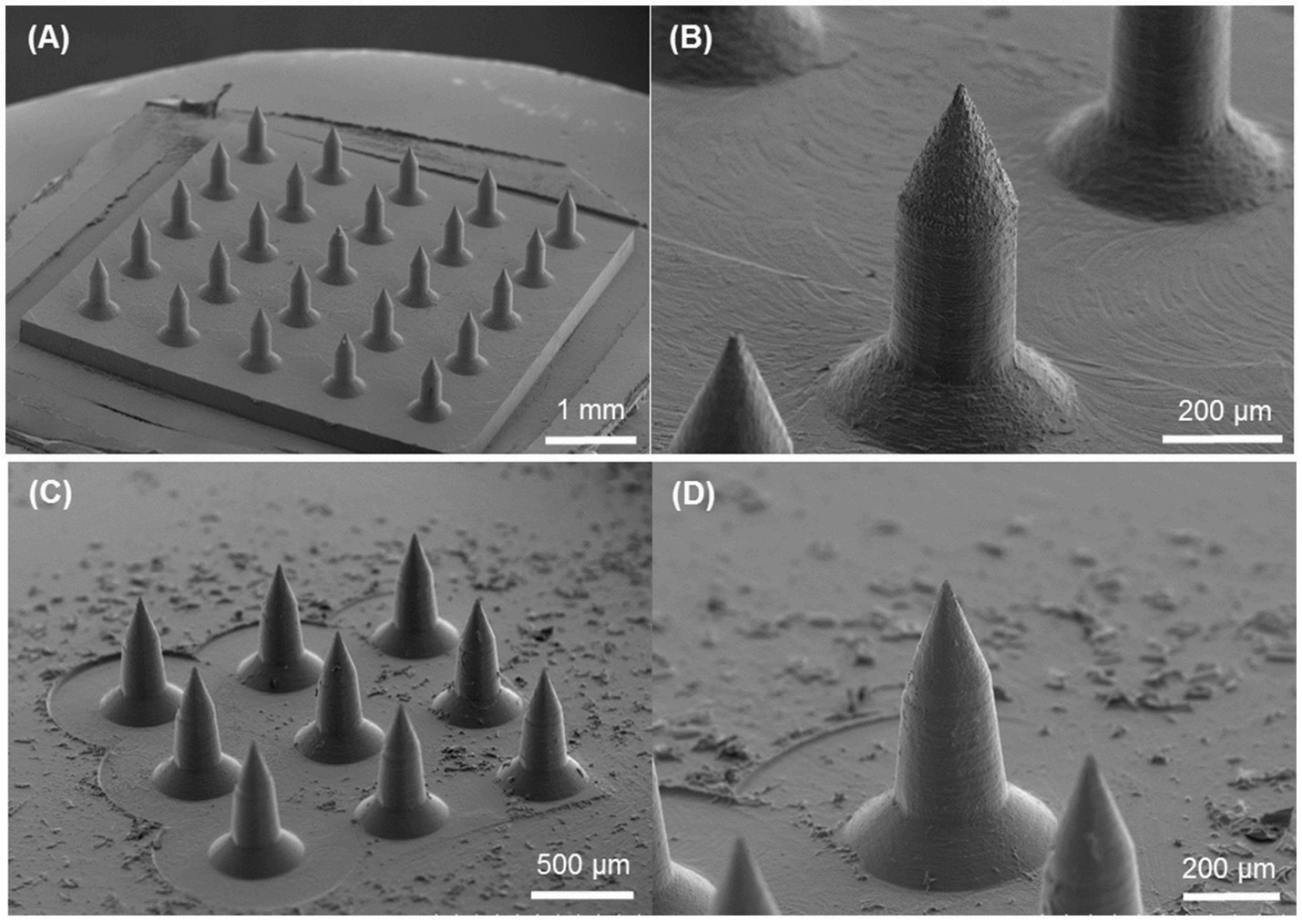
SEM images of PLA masters after oxygen plasma etching. **(A)** 5 × 5 array of circular obelisk microneedles. **(B)** Close-up view of circular obelisk. **(C)** 3 × 3 array of beveled-circular obelisk microneedles. **(D)** Close-up view of beveled-circular obelisk.

Circular obelisk-type geometries having a truncated cone base were designed for facilitating the spray deposition process; a curved surface prevents undesirable deposition along the edges of a mold, which often occurs when a polyhedral mold (e.g., pyramidal microneedle mold) is used for spray deposition. Also, the truncated base structure helps funnel the sprayed droplets into the mold cavity. Circular and beveled-circular obelisk microneedle master structures were successfully fabricated by a micro-milling process followed by micromolding and plasma etching. Figure 3 shows the scanning electron microscope (SEM) images of the circular obelisk (Figures 3A,B) and the beveled-circular obelisk (Figures 3C,D) PLA microneedles. The detailed dimensions of each microneedle structure as represented in Figure 2B were measured using the SEM images and summarized in Table 2. For the circular obelisk, the height and width were approximately 420.69 ± 2.93 μm and 214.22 ± 2.53 μm, respectively. The beveled-circular obelisk was slightly larger in both dimensions, approximately 455.41 ± 6.21 μm in height and 240.79 ± 6.95 μm in width. Both microneedle arrays had almost identical center-to-center spacing between needles (592.96 ± 21.81 μm for circular obelisk and 599.34 ± 19.66 μm for beveled-circular obelisk). The aspect ratio (height/width) of two geometries was similar to each other (1.96 for circular obelisk, 1.89 for beveled-circular obelisk). In addition, the PLA microneedles replicated from the aluminum primary master showed slight volumetric shrinkage: 9.02 ± 4.23% and 5.64 ± 2.84% for circular obelisk and beveled-circular obelisk geometries, respectively. The dimensions of the aluminum primary masters were provided in Supplementary Table 1. After oxygen plasma etching, the diameter of microneedle tips decreased from 15.83 ± 3.17 to 7.41 ± 1.48 μm for circular obelisk needle geometry, and from 13.99 ± 0.80 to 6.64 ± 1.71 μm for beveled-circular obelisk geometry (Supplementary Figure 1), which would enhance skin penetration capability of microneedles. The plasma-etched PLA masters were used to prepare PDMS molds for microneedle fabrication.

**Table 2:** Dimensions of PLA masters measured from SEM images (mean ± standard deviation, *n* = 7).

**Microneedle geometry**	**Apex angle (degrees, α_apex_)**	**Bevel angle (degrees, α_bevel_)**	**Diameter (**μ**m)**			**Height (**μ**m)**			**Volume Shrinkage (%)**
			**D_tip_**	**D_stem_**	**D_base_**	**H_tip_**	**H_stem_**	**H_base_**	
Circular obelisk	56.66 ± 1.27	89.30 ± 0.24	214.22 ± 2.53	214.22 ± 2.53	434.78 ± 3.33	198.74 ± 5.54	221.95 ± 6.20	62.74 ± 2.37	9.02 ± 4.23
Beveled-circular obelisk	49.71 ± 0.84	83.70 ± 0.65	184.27 ± 2.01	240.79 ± 6.95	445.95 ± 4.70	198.99 ± 5.59	256.42 ± 7.87	89.90 ± 4.72	5.64 ± 2.84

### Fabrication of polymer microneedles using spray deposition

**Figure 4:**
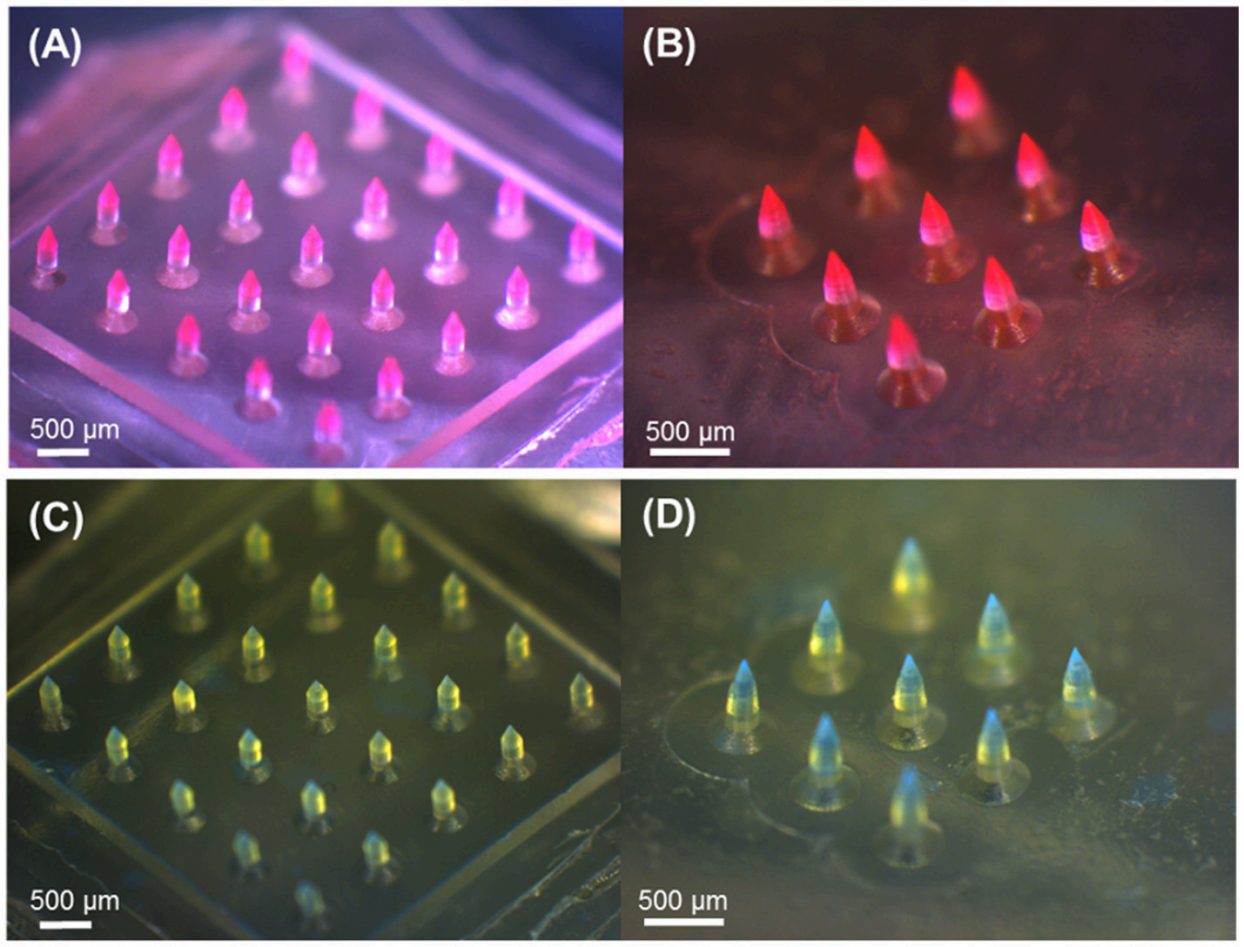
Optical microscopy images of obelisk-type polymer microneedles fabricated by the spray deposition process. PVP and PLGA layers contain SRB (red) and coumarin 314 (blue), respectively, for visualization. **(A)** PVP circular obelisk microneedle array. **(B)** PVP beveled-circular obelisk microneedle array. **(C)** PLGA circular obelisk microneedle array. **(D)** PLGA beveled-circular obelisk microneedle array. Scale bars represent 500 μm.

We firstly aimed to fabricate obelisk-type microneedles from a single material (PVP or PLGA) using the spray deposition process to identify proper spraying parameters. Unlike conventional micromolding techniques, which often require highly concentrated formulations and/or harsh processing conditions such as high temperature and centrifugation, the spray deposition process adopted in this study utilizes polymer solutions at low concentrations and mild drying conditions, thereby potentially being beneficial to improve the stability of labile substances incorporated in microneedles (Donnelly et al., 2009). With the specific spray parameters and solution formulation summarized in Table 1, both circular and beveled-circular obelisk microneedles were successfully fabricated from PVP and PLGA (Figure 4). As shown in Figures 4A,B SRB dye was localized at the tip of microneedles since the fine droplets of PVP aqueous solution were rolled on the hydrophobic PDMS mold surface upon spraying and accumulated into the mold cavity before solidification. Figures 4C,D display the PLGA microneedles containing coumarin 314 at the needle tips. The dimensions of the fabricated PVP and PLGA microneedles were measured based on their SEM images (images not shown) and were summarized in Tables 3, 4, respectively. In each case, the apex and bevel angles of the PVP and PLGA microneedles were observed to be smaller than those of the PLA masters. The diameters and heights were also reduced due to volume shrinkage during solvent evaporation. It was observed that the circular obelisk geometry showed average shrinkage of 13.22% (average of PVP and PLGA microneedles), whereas the beveled-circular obelisk geometry showed the average shrinkage of 18.29%.

**Table 3:** Measured dimensions of PVP microneedles (mean ± standard deviation, *n* = 7).

**Microneedle geometry**	**Apex angle (degree, α_apex_)**	**Bevel angle (degree, α_bevel_)**	**Diameter (**μ**m)**			**Height (**μ**m)**			**Volume shrinkage (%)**
			**D_tip_**	**D_stem_**	**D_base_**	**H_tip_**	**H_stem_**	**H_base_**	
Circular obelisk	54.69 ± 1.05	88.14 ± 0.61	202.49 ± 4.86	202.49 ± 4.86	426.01 ± 5.88	195.77 ± 2.34	210.94 ± 5.33	61.45 ± 8.42	12.57 ± 4.72
Beveled-circular obelisk	45.84 ± 2.02	83.34 ± 0.72	160.90 ± 9.82	226.08 ± 3.27	430.56 ± 2.56	194.55 ± 3.88	267.21 ± 8.66	60.70 ± 7.83	17.42 ± 6.65

**Table 4:** Measured dimensions of PLGA microneedles (mean ± standard deviation, *n* = 7).

**Microneedle geometry**	**Apex angle (degree, α_apex_)**	**Bevel angle (degree, α_bevel_)**	**Diameter (**μ**m)**			**Height (**μ**m)**			**Volume shrinkage (%)**
			**D_tip_**	**D_stem_**	**D_base_**	**H_tip_**	**H_stem_**	**H_base_**	
Circular obelisk	54.92 ± 1.91	88.52 ± 0.42	200.46 ± 1.60	200.46 ± 1.60	400.80 ± 11.87	193.06 ± 8.09	200.36 ± 6.38	58.47 ± 8.72	13.86 ± 4.28
Beveled-circular obelisk	44.34 ± 1.09	82.92 ± 0.76	168.67 ± 2.49	233.22 ± 7.35	427.50 ± 2.69	195.04 ± 4.32	259.97 ± 1.93	64.27 ± 4.48	19.16 ± 5.72

**Figure 5:**
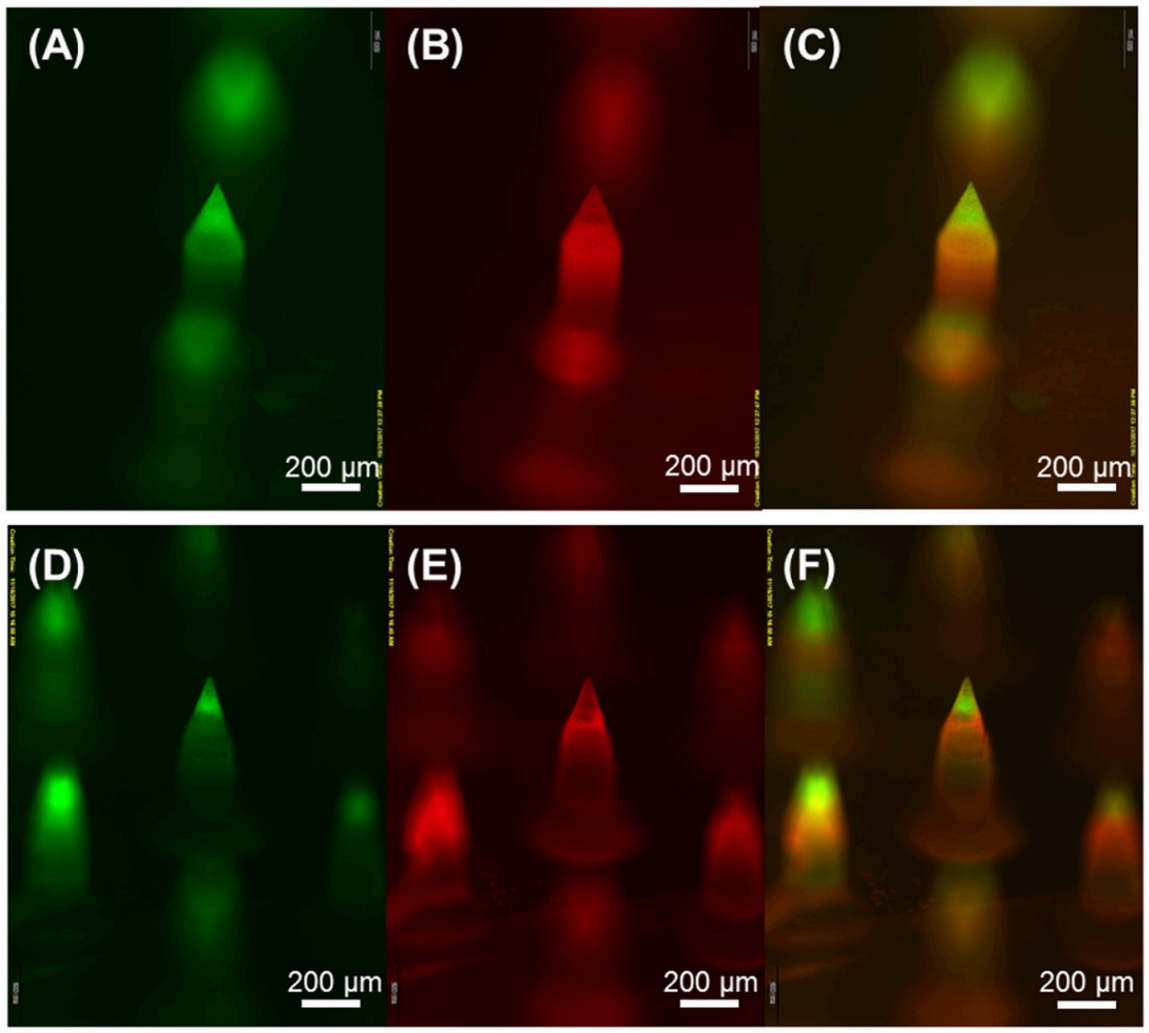
Fluorescence microscopy images of horizontally layered microneedles. Green and red fluorescence corresponds to coumarin 314-containing PLGA and SRB-containing PVP layer, respectively. **(A,D)** show PLGA microneedle tips, **(B,E)** show PVP needle shafts, and **(C,F)** are overlay images. Scale bars represent 200 μm.

Based on the spraying parameters and solution concentrations identified from the monolithic microneedle fabrication, we next attempted to fabricate horizontally layered microneedles with heterogeneous compositions. The fabricated microneedles were composed of two horizontal layers, biodegradable PLGA and water-soluble PVP (MW 10 KDa) layers, with a backing layer (40 kDa PVP+2.5% glycerol). For clear visualization of each layer, coumarin 314 (green) fluorescence dye and SRB (red) dye were added to PLGA and PVP solutions, respectively. Figure 5 shows the fabricated multilayer microneedles with PLGA tips (Figures 5A,D) and PVP shafts (Figures 5B,E). The overlay images (Figures 5C,F) demonstrate that horizontal layers were successfully formed by sequential spray deposition of PLGA and PVA solutions for both geometries.

### Microneedle insertion into porcine skin

**Figure 6:**
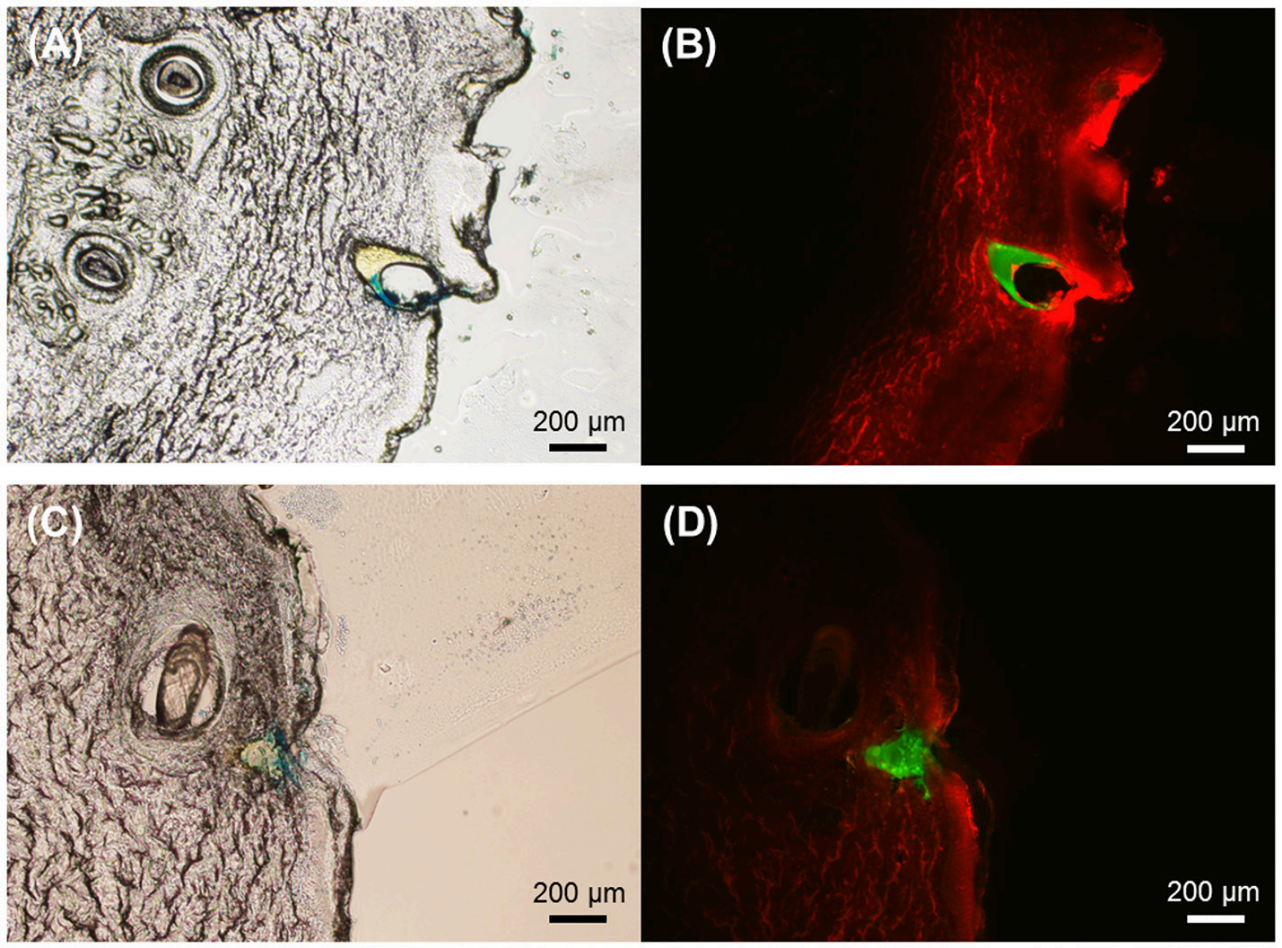
Representative cross-sectional images of pig cadaver skin after insertion of PLGA-PVP multilayer microneedles. **(A)** The brightfield image and **(B)** The corresponding fluorescence image of the skin after insertion of circular obelisk microneedles. **(C)** The brightfield image and **(D)** The corresponding fluorescence image of the skin after insertion of beveled-circular obelisk microneedles. Fluorescence images show that the PLGA tips (green) remain intact in the skin while the PVP shafts (red) are completely dissolved. Scale bars represent 200 μm.

The fabricated multilayer microneedles were tested for skin insertion capability using pig cadaver skin. The microneedles were pressed onto the skin with thumb pressure (approximately 3–5 N/cm^2^, Ryu et al., 2018) and held in place for 5 min. The microneedle insertion sites stained after microneedle removal (Supplementary Figure 2) confirmed that all microneedles were successfully inserted into the skin. Figure 6 displays the cross-sectional view of the skin insertion sites for both microneedle geometries. As shown in Figures 6B,D SRB in the PVP layer spread widely in the skin, whereas coumarin 314 in the PLGA layer seemed not to diffuse within the given time frame. This result indicates that the PVP layer was quickly dissolved within 5 min of application time, which facilitates the separation of the PLGA tips from the PVP shaft, resulting in the embedment of the PLGA tips in the skin. The PLGA layer could act as a drug depot for sustained drug release. Therefore, the multilayer microneedles have great potential applications in implantable devices for controlled drug delivery.

### Mechanical stability of microneedles

**Figure 7:**
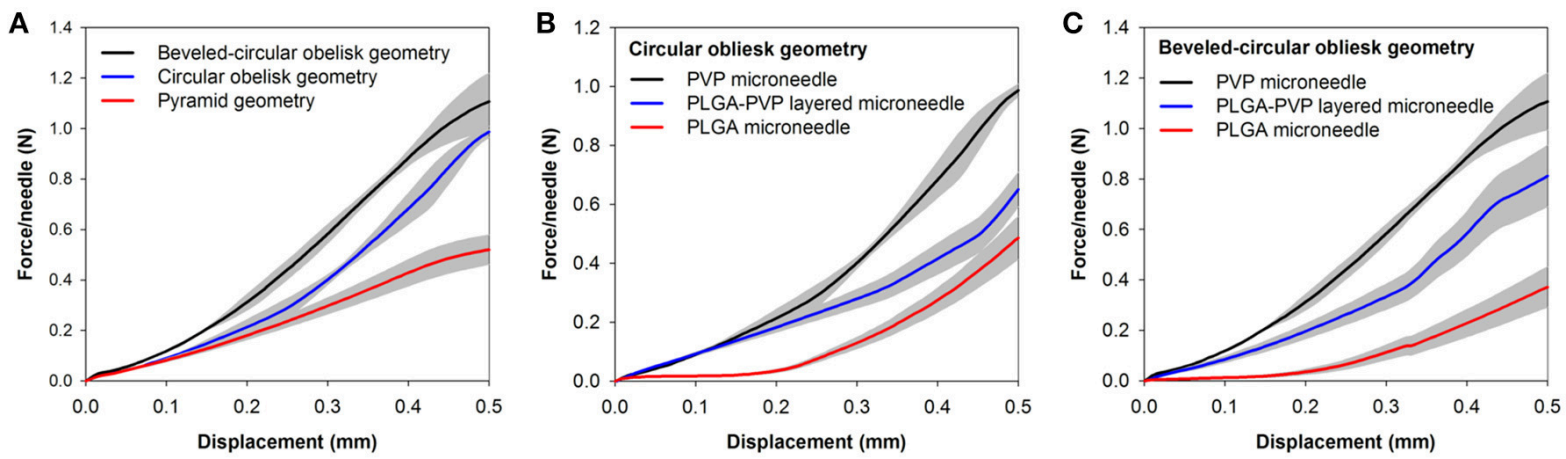
Force-displacement curves of microneedles. **(A)** Mechanical behavior of PVP microneedles with different geometries. Square pyramid microneedles (600 μm height and 300 μm base) were measured for comparison. **(B)** Mechanical behavior of circular obelisks with different material compositions. **(C)** Mechanical behavior of beveled-circular obelisks with different material compositions. Data represent the averaged value of 5 independent measurements with standard deviation (shadow zone).

We further investigated the mechanical behavior of the obelisk-type microneedles under compression. Together with the two geometries, conventional pyramidal-shaped microneedles (300 μm square base, 600 μm height) were tested for comparison. Figure 7A exhibits the force-displacement curves for PVP microneedles with different geometries. All the curves from three different geometries did not show a distinct transition point that indicates buckling failure over the experimental ranges, which is consistent with the findings of other researchers (Lee et al., 2008; Xu et al., 2017). However, each geometry showed different mechanical behaviors over the displacement ranges. Since the microneedles tested are composed of the same material (PVP), the differences in mechanical behavior should be attributed to the microneedle geometry. It was found that the beveled-circular obelisk shape possessed the greatest resistance against the compressive force, which was approximately 2.12 times higher than that of pyramid geometry required for 0.5 mm displacement. The circular obelisk microneedle showed slight lower stiffness than the beveled-circular one but possessed significantly higher stiffness than the pyramid. Our results demonstrate that the mechanical stability of the obelisk-type geometries designed in this work is substantially greater than that of pyramidal geometry which has been widely used in microneedle designs.

To further study the mechanical behavior of the heterogeneously layered microneedle, PLGA-PVP microneedles were tested and compared with single-layer microneedles (i.e., PLGA-only and PVP-only microneedles). Figures 7B,C show the force-displacement curves of the circular obelisk and beveled-circular obelisk microneedles with different layer compositions, respectively. For both geometries, PVP microneedles showed the highest mechanical stability against axial compression, which was over 2.5 times higher than PLGA microneedles. Also, it was found that the mechanical stiffness of PLGA-PVP microneedles was intermediate between that of PLGA and PVP microneedles. This result was anticipated since the layered microneedles are a composite of PVP and PLGA. More importantly, there was no sudden force drop observed, indicating that mechanical failure at the interface between two layers did not occur during the compression test and representing that strong adhesion between two layers was successfully achieved.

### Influence of spray deposition on the integrity of horseradish peroxidase (HRP)

We chose a spraying method for microneedle fabrication, assuming that mild processing conditions of the spray deposition would reduce the loss of drug potency during fabrication. In this study, HRP was used as a model protein drug, and its secondary structure and enzymatic activity during encapsulation in PVP and PLGA polymer matrices were measured by CD and colorimetric assay, respectively. To obtain the sufficient amount of HRP for analyses, we collected samples on a 10 cm diameter circular PDMS substrate that mimics the surface of PDMS microneedle molds.

**Figure 8:**
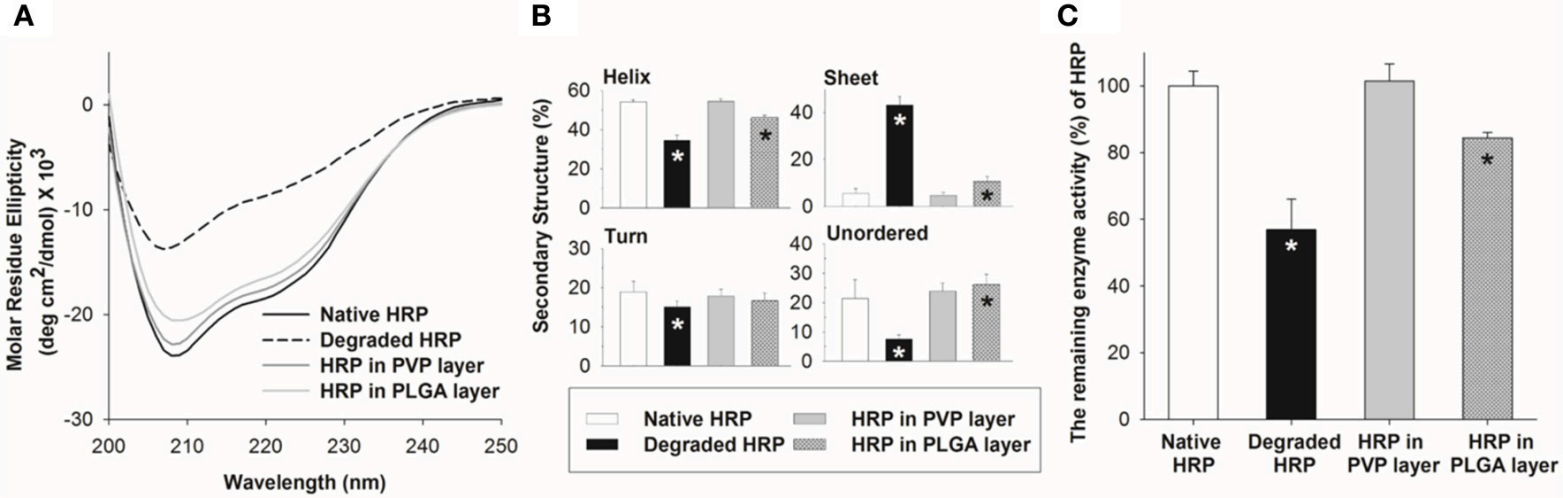
Stability of HRP in polymer matrices upon spray deposition. **(A)** CD spectra of HRP in PVP and PLGA matrix. **(B)** Secondary structure compositions of HRP estimated from the CD spectra by the CDSSTR algorithm. **(C)** Relative enzyme activity of HRP in PVP and PLGA matrix. Asterisk (*) indicates a significant difference between a processed HRP sample and native HRP (*n* = 3, *p* < 0.05).

Figure 8A shows the CD spectra of HRP encapsulated in the PVP and PLGA matrices. Untreated (native) and acid-treated HRP were used as controls. The untreated HRP had two negative ellipticity values at 208 and 222 nm and contained 54.4% α-helix, 6.4% β-sheet, 20.5% turn and 18.6% unordered structures. On the other hand, the degraded HRP samples (acid-treated) showed a significant decrease in ellipticity values and slight peak shift. In addition, α-helix content of the degraded HRP was significantly decreased compared with native HRP, along with an increase of sheet, turn and unordered structures due to the disruption of the secondary structure caused by acid treatment. The CD spectrum of HRP in the PVP layer showed no detectable changes in the secondary structure compared to native HRP, whereas there was a noticeable change in the CD spectrum of HRP encapsulated in the PLGA layer. The estimated secondary structural composition of HRP revealed that HRP in PVP retained its structural stability while the secondary structure distribution of HRP in PLGA was significantly different from that of untreated HRP (*p* < 0.05) as shown in Figure 8B.

To further examine the HRP structural integrity, the enzymatic activity of HRP encapsulated in the polymer matrices was measured and compared with that of untreated and acid-treated HRP samples. The relative enzyme activity of HRP in the PVP layer was retained without any loss, but HRP in the PLGA layer showed approximately 85% enzyme activity. This result suggests that the use of organic solvent and emulsification required to prepare HRP containing PLGA solution could lower the stability of HRP.

## Discussion

In this study, micro-milling technique was utilized to fabricate obelisk-type microneedle masters. Conventional microfabrication techniques, such as photolithography and etching, have been widely used to form microneedles from silicon, silicon-oxides, photoresist and metals, but the creation of complex geometries requires multiple processing steps and high-cost equipment. Recently, micro-milling has been introduced as an alternative approach for fabricating microneedle masters (Bediz et al., 2014; Korkmaz et al., 2015) due to its advantages over conventional approaches, including simple design process and low-cost manufacturing for generating complex geometries with high accuracy and repeatability. Furthermore, micro-milling does not require the use of highly toxic chemicals, allowing safe and eco-friendly production of microstructures.

Since micro-milling is performed under very high spindle speed, it is critical to select appropriate materials for machining. Milling materials should bear suitable hardness and good wear resistance to produce sharp, micrometer-scale structures accurately. Also, the material should be thermally stable because high-speed friction often deforms or melts small structures during milling. In this study, aluminum 6061 alloy was used for fabricating primary masters due to its good machinability, mechanical strength, and thermal stability. Although metal masters were successfully fabricated by micro-milling, SEM observation revealed that the dimensions of the masters were quite different from the original designs. For example, the stem diameter of the beveled-circular obelisk was designed to be 250 μm, but the measured diameter was approximately 243 μm. It was also not easy to achieve extremely sharp tips (smaller than 10 μm diameter) because tips were easily deformed due to excessive heat during milling. Therefore, those limitations should be considered during the device design phase. Once the primary masters were formed by micro-milling, we prepared secondary PLA masters through successive micromolding processes to further sharpen the tips of microneedles. Previously, we attempted to sharpen the tips of aluminum masters by chemical etching, but it was not easy to control the etching rate and profile; master structures should be prepared repeatedly until the etch process is optimized. Therefore, multiple PLA structures were replicated from the aluminum masters and subsequently subjected to oxygen plasma etching for sharpening tips. The plasma system used for etching showed very slow etch rates (approximately 4 μm/h) since it is originally designed for surface treatment (i.e., low frequency, low power). The etching time would be significantly reduced when high frequency plasma is used.

In the past decades, polymer microneedles have been fabricated typically by the micromolding process in which a drug-polymer solution or polymer molten is filled into a mold and solidified with the application of centrifugation, vacuum or high temperature (Donnelly et al., 2012; Larraneta et al., 2016). To achieve complete filling of materials into a mold with less drying time, highly concentrated solutions are preferred for the micromolding process. However, those processing conditions could cause instability of sensitive molecules during microneedle fabrication, leading to the loss of drug potency. As an alternative, the spray deposition process was recently introduced to form polymer microneedles from various water-soluble polymers, including sugars, PVA and CMC (McGrath et al., 2014). Our group further reported a new fabrication technique, the dual-nozzle spray deposition process, which utilizes a separate deposition of drug and polymer solutions, as a potential way to address the stability issue associated with current microneedle fabrication processes (Kim et al., 2017), and demonstrated that the spray deposition process could improve the retention of structural stability of BSA during microneedle fabrication compared to the vacuum-assisted micromolding process. Based on the previous studies, we anticipated that the spray deposition process could be used for fabricating multilayer microneedles, which are composed of biodegradable and water-soluble polymer layers while minimally affecting the stability of labile molecules, especially protein-based drugs.

The results shown in Figures 4, 5 clearly demonstrate that the spray deposition process is capable of forming both single layer and multilayer microneedles from PVP and PLGA. Particularly, the model drugs (dyes) were only loaded in the tip of the microneedles (Figure 4), which can permit efficient drug delivery into the skin with high dosing accuracy while minimizing undesirable drug encapsulation in the backing layer. Figure 6 confirms that the PLGA tips containing a model drug were completely embedded in the skin without leaving any observable traces of drugs on the skin. Fluorescence microscopy images of the multilayer microneedles also showed clear formation of horizontal layers by sequential deposition of PLGA and PVP (Figure 5). Due to the high surface tension and low vapor pressure of aqueous PVP solutions, the sprayed PVP droplets were rolled into a fine cavity of the hydrophobic PDMS mold and solidified. To form a horizontal PLGA layer, it was necessary to modulate solvent evaporation rate and spraying parameters. When ethyl acetate was used as a solvent of PLGA, only conformal film was formed on the mold surface regardless of spraying conditions due to fast evaporation of the solvent (Kim et al., 2017). In this study, a mixture of ethyl acetate and dimethyl carbonate (1:4 v/v) was used to retard solvent evaporation, and the formation of horizontal PLGA layers was achieved under high flow rate and low spraying pressure. It is anticipated that the deposition profile of polymer solutions could be modulated by adjusting solvent compositions and spraying parameters. We also estimated the volume shrinkage of each microneedle by comparing the dimensions of the fabricated microneedles with those of the PLA masters. It was observed that the shrinkage rate of PLGA microneedles was higher than that of PVP microneedles, which may be due to the concentration of PLGA in the solution (1% w/v) was lower than that of PVP (5% w/v).

As discussed above, we demonstrated the fabrication of layered microneedles with heterogeneous compositions, which have potential applications in implantable drug delivery systems. As shown in Figure 6, the first PLGA layer of the microneedles was fully embedded in the skin, while the second PVP layer was quickly dissolved within 5 min after insertion, indicating that the PLGA-PVP layer composition could be capable of producing a biphasic release profile. We anticipate that drug release profiles could be readily controlled by adjusting various parameters, including material combinations, ratio of copolymers, molecular weight, functional groups and drug amounts in each layer. We also expect that the spray deposition process could be a time- and cost-effective approach to produce heterogeneously layered, multidrug-containing microneedles, which have potential applications in delivering multivalent vaccines in a controlled manner. In addition, the multilayer microneedles could be an attractive approach for insulin delivery, since biphasic delivery of insulin is desirable to maintain insulin levels while reducing administration frequency.

Mechanical stability of microneedles is essential for achieving reliable skin penetration. Our mechanical testing data shown in Figure 7 strongly demonstrate that obelisk-type microneedles possess better mechanical stiffness than pyramidal microneedles. Since all the microneedles tested have almost identical aspect ratios (approximately 2), higher mechanical stiffness of the obelisk-type microneedles is attributed to larger cross-sectional area at a given height compared to pyramidal microneedles. When the same compression force is applied to microneedles, obelisks experience less compressive stress than pyramids per unit cross-sectional area, resulting in less deformation. When comparing two obelisk microneedles, the beveled-circular obelisks have slightly larger cross-sectional area than the circular obelisks, resulting in more resistance against compression. This result further indicates that the obelisk-type microneedles could be advantageous in terms of drug loading capacity due to larger microneedle volume per given aspect ratio compared to conventional pyramidal or conical microneedles. Our data also suggest that the adhesion between PLGA and PVP layers was strong enough to resist breaking at the layer interface. We anticipate that adhesive properties of PVP facilitate the successful binding between two layers (Lee, 2005; Teodorescu and Bercea, 2015).

To evaluate the integrity of drugs upon spray deposition, we chose HRP as a model protein drug. HRP, found in the roots of horseradish, has been extensively used in various applications including diagnostics, bio-sensing and cancer therapy (Veitch, 2004; Gupta et al., 2016; Li et al., 2017). Since the conformational changes of HRP are highly related to its function, we investigated the structural changes and enzyme activity of HRP during the spray deposition process. The secondary structure of HRP was investigated using CD spectroscopy, and the enzyme activity was measured via a reaction with 3,3′,5,5′-tetramethylbenzidine (TMB), a commonly used chromogen for HRP. The mechanism of oxidation of TMB by HRP is a well-known process. Briefly, oxidation of TMB by HRP/H_2_O_2_ first generates a blue-colored complex product, which turns yellow after the addition of phosphoric acid to the reaction media. The activity of HRP is then determined by measuring the absorbance at 450 nm. We found that HRP encapsulated in the PVP polymer matrix retained its secondary structure and enzymatic function as shown in Figure 8. This result is consistent with our previous findings, indicating that mild processing conditions, such as fast drying at ambient temperature and no pressure/vacuum application, would contribute to the maintenance of enzyme stability (Kim et al., 2017). This also suggests that the air-water interfacial stress due to the large surface-to-volume ratio of the sprayed droplets has a marginal effect on the structural stability of HRP. However, HRP encapsulated in the PLGA polymer matrix lost its structural stability to a certain degree (Figure 8), which might be caused by the emulsification process. Emulsification and homogenization are known to disturb the conformation of proteins and trigger various physicochemical changes at the water-organic phase interface, resulting in losing therapeutic activity or causing unpredictable side effects (Sah, 1999; van de Weert et al., 2000; Bilati et al., 2005). This finding was also comparable with our previous discovery that BSA emulsified in PLGA solution induced a partial loss of the secondary and tertiary structures (Kim et al., 2017).

## Conclusions

Obelisk-type multilayer polymer microneedles were successfully fabricated by micro-milling and spray deposition. Micro-milling was capable of forming complex micro-scale structures with an accurate and reproducible fashion. Two geometries, circular obelisk and beveled-circular obelisk, were fabricated from aluminum alloy and replicated in PLA. The tips of PLA microneedles were sharpened by oxygen plasma. The spray deposition process demonstrated the formation of polymer microneedles from PVP and PLGA. The spray-formed microneedles showed different volumetric shrinkage rates depending on solution concentrations. By modulating solution compositions and spraying parameters, multipayer PLGA-PVP microneedles were successfully fabricated. Histologic examination demonstrated that the multilayer microneedles released dyes into the skin at different rates. Mechanical testing showed that the obelisk-shaped microneedles have higher mechanical stiffness compared to a pyramid-shaped microneedle, and adhesion between PLGA and PVP layers was strong enough to hold the layers together during compression. The structural integrity and catalytic efficiency of HRP were well-maintained in PVP, whereas HRP in PLGA showed structural changes and decreased enzyme activity upon the spray deposition process. In conclusion, the combination of micro-milling and spray deposition would be a versatile method to produce polymer microneedles with various geometries, and multilayer microneedles would have potential applications in controlled-release implantable drug delivery devices.

## Author contributions

MK and SP: designed and conducted the experiments and wrote the first draft of the manuscript; BR, GG, and S-KB: contributed design and fabrication of microneedle masters; MK, SP, and S-OC: analyzed and interpreted the data, and revised the manuscript; J-HP, AB, and S-OC: conceived and supervised the project. All authors read and approved the manuscript.

### Conflict of interest statement

S-KB was employed by QuadMedicine Co., Ltd. The remaining authors declare that the research was conducted in the absence of any commercial or financial relationships that could be construed as a potential conflict of interest.

## References

[B1] Aoyagi S. Izumi H. Isono Y. Fukuda M. Ogawa H. (2007). Laser fabrication of high aspect ratio thin holes on biodegradable polymer and its application to a microneedle. Sens. Actuat. A 139, 293–302. 10.1016/j.sna.2006.11.022

[B2] ASTM Standard D695-15 (2015). Standard Test Method for Compressive Properties of Rigid Plastics. West Conshohocken: ASTM International 08.01.

[B3] Bediz B. Korkmaz E. Khilwani R. Donahue C. Erdos G. Falo L. D. Jr.. (2014). Dissolvable microneedle arrays for intradermal delivery of biologics: fabrication and application. Pharm. Res. 31, 117–135. 10.1007/s11095-013-1137-x 23904139 PMC3898465

[B4] Bilati U. Allemann E. Doelker E. (2005). Strategic approaches for overcoming peptide and protein instability within biodegradable nano- and microparticles. Eur. J. Pharm. Biopharm. 59, 375–388. 10.1016/j.ejpb.2004.10.006 15760718

[B5] Chu L. Y. Choi S.-O. Prausnitz M. R. (2010). Fabrication of dissolving polymer microneedles for controlled drug encapsulation and delivery: bubble and pedestal microneedle designs. J. Pharm. Sci. 99, 4228–4238. 10.1002/jps.22140 20737630

[B6] Donnelly R. F. Morrow D. I. J. Singh T. R. R. Migalska K. McCarron P. A. O'Mahony C.. (2009). Processing difficulties and instability of carbohydrate microneedle arrays. Drug Dev. Ind. Pharm. 35, 1242–1254. 10.1080/03639040902882280 19555249 PMC2900182

[B7] Donnelly R. F. Singh T. R. R. Morrow D. I. J. Woolfson A. D. (2012). Microneedle-Mediated Transdermal and Intradermal Drug Delivery. London, UK: John Wiley & Sons, Ltd.

[B8] Gomaa Y. A. Garland M. J. McInnes F. El-Khordagui L. K. Wilson C. Donnelly R. F. (2012). Laser-engineered dissolving microneedles for active transdermal delivery of nadroparin calcium. Eur. J. Pharm. Biopharm. 82, 299–307. 10.1016/j.ejpb.2012.07.008 22836025 PMC4119960

[B9] Gupta N. Gupta C. Sharma S. Rathi B. Sharma R. K. Bohidar H. B. (2016). Magnetic iron oxide nanoparticles encapsulating horseradish peroxidase (HRP): synthesis, characterization and carrier for the generation of free radicals for potential applications in cancer therapy. RSC Adv. 6, 111099–111108. 10.1039/C6RA24586B

[B10] Henry S. McAllister D. Allen M. G. Prausnitz M. R. (1998). Microfabricated microneedles: a novel approach to transdermal drug delivery. J. Pharm. Sci. 87, 922–925. 10.1021/js980042+ 9687334

[B11] Hirobe S. Azukizawa H. Hanafusa T. Matsuo K. Quan Y. Kamiyama F.. (2015). Clinical study and stability assessment of a novel transcutaneous influenza vaccination using a dissolving microneedle patch. Biomaterials 57, 50–58. 10.1016/j.biomaterials.2015.04.007 25913250

[B12] Hong X. Wei L. Wu F. Wu Z. Chen L. Liu Z.. (2013). Dissolving and biodegradable microneedle technologies for transdermal sustained delivery of drug and vaccine. Drug Des. Devel. Ther. 7, 945–952. 10.2147/DDDT.S44401 24039404 PMC3771849

[B13] Indermun S. Luttge R. Choonara Y. E. Kumar P. du Toit L. C. Modi G.. (2014). Current advances in the fabrication of microneedles for transdermal delivery. J. Control. Release 185, 130–138. 10.1016/j.jconrel.2014.04.052 24806483

[B14] Kim J. D. Kim M. Yang H. Lee K. Jung H. (2013). Droplet-born air blowing: novel dissolving microneedle fabrication. J. Control. Release 170, 430–436. 10.1016/j.jconrel.2013.05.026 23742882

[B15] Kim M. J. Park S. C. Choi S.-O. (2017). Dual-nozzle spray deposition process for improving the stability of proteins in polymer microneedles. RSC Adv. 7, 55350–55359. 10.1039/C7RA10928H

[B16] Kim Y.-C. Park J.-H. Prausnitz M. R. (2012). Microneedles for drug and vaccine delivery. Adv. Drug Deliv. Rev. 64, 1547–1568. 10.1016/j.addr.2012.04.005 22575858 PMC3419303

[B17] Korkmaz E. Friedrich E. E. Ramadan M. H. Erdos G. Mathers A. R. Ozdoganlar O. B.. (2015). Therapeutic intradermal delivery of tumor necrosis factor-alpha antibodies using tip-loaded dissolvable microneedle arrays. Mater. Sci. Eng. R Rep. 24, 96–105. 10.1016/j.actbio.2015.05.036 26093066 PMC8266287

[B18] Larraneta E. Lutton R. E. M. Woolfson A. D. Donnelly R. F. (2016). Microneedle arrays as transdermal and intradermal drug delivery systems: materials science, manufacture and commercial development. Mater. Sci. Eng. R 104, 1–32. 10.1016/j.mser.2016.03.001

[B19] Lee J. (2005). Intrinsic adhesion properties of poly(vinyl pyrrolidone) to pharmaceutical materials: humidity effect. Macromol. Biosci 5, 1085–1093. 10.1002/mabi.200500146 16245267

[B20] Lee J. W. Park J.-H. Prausnitz M. R. (2008). Dissolving microneedles for transdermal drug delivery. Biomaterials 29, 2113–2124. 10.1016/j.biomaterials.2007.12.048 18261792 PMC2293299

[B21] Li H. Chang J. Hou T. Li F. (2017). HRP-mimicking DNAzyme-Catalyzed *in situ* generation of polyaniline to assist signal amplification for ultrasensitive surface plasmon resonance biosensing. Anal. Chem. 89, 673–680. 10.1021/acs.analchem.6b02988 28105837

[B22] Lobley A. Whitmore L. Wallace B. (2002). DICHROWEB: an interactive website for the analysis of protein secondary structure from circular dichroism spectra. Bioinformatics 18, 211–212. 10.1093/bioinformatics/18.1.211 11836237

[B23] McGrath M. G. Vucen S. Vrdoljak A. Kelly A. O'Mahony C. Crean A. M.. (2014). Production of dissolvable microneedles using an atomised spray process: effect of microneedle composition on skin penetration. Eur. J. Pharm. Biopharm. 86, 200–211. 10.1016/j.ejpb.2013.04.023 23727511

[B24] Mistilis M. J. Bommarius A. S. Prausnitz M. R. (2015). Development of a thermostable microneedle patch for influenza vaccination. J. Pharm. Sci. 104, 740–749. 10.1002/jps.24283 25448542 PMC5750137

[B25] Miyano T. Tobinaga Y. Kanno T. Matsuzaki Y. Takeda H. Wakui M.. (2005). Sugar micro needles as transdermic drug delivery system. Biomed. Microdevices 7, 185–188. 10.1007/s10544-005-3024-7 16133805

[B26] Park J.-H. Allen M. G. Prausnitz M. R. (2005). Biodegradable polymer microneedles: fabrication, mechanics and transdermal drug delivery. J. Control. Release 104, 51–66. 10.1016/j.jconrel.2005.02.002 15866334

[B27] Park J. H. Allen M. G. Prausnitz M. R. (2006). Polymer microneedles for controlled-release drug delivery. Pharm. Res. 23, 1008–1019. 10.1007/s11095-006-0028-9 16715391

[B28] Perennes F. Marmiroli B. Matteucci M. Tormen M. Vaccari L. Di Fabrizio E. (2006). Sharp beveled tip hollow microneedle arrays fabricated by LIGA and 3D soft lithography with polyvinyl alcohol. J. Micromech. Microeng. 16, 473–479. 10.1088/0960-1317/16/3/001

[B29] Prausnitz M. R. (2017). Engineering microneedle patches for vaccination and drug delivery to skin. Annu. Rev. Chem. Biomol. Eng. 8, 177–200. 10.1146/annurev-chembioeng-060816-101514 28375775

[B30] Quinn H. L. Kearney M. Courtenay A. J. McCrudden M. T. C. Donnelly R. F. (2014). The role of microneedles for drug and vaccine delivery. Expert Opin. Drug Delivery 11, 1769–1780. 10.1517/17425247.2014.938635 25020088

[B31] Rouphael N. G. Paine M. Mosley R. Henry S. McAllister D. V. Kalluri H.. (2017). The safety, immunogenicity, and acceptability of inactivated influenza vaccine delivered by microneedle patch (TIV-MNP 2015): a randomised, partly blinded, placebo-controlled, phase 1 trial. Lancet 390, 649–658. 10.1016/S0140-6736(17)30575-5 28666680 PMC5578828

[B32] Ryu H. R. Jeong H. R. Seon-Woo H. Kim J. S. Lee S. K. Kim H. J.. (2018). Efficacy of a bleomycin microneedle patch for the treatment of warts. Drug Delivery Transl. Res. 8, 273–280. 10.1007/s13346-017-0458-4 29204924

[B33] Sah H. (1999). Protein behavior at the water/methylene chloride interface. J. Pharm. Sci. 88, 1320–1325. 10.1021/js9900654 10585229

[B34] Sreerama N. Venyaminov S. Woody R. (2000). Estimation of protein secondary structure from circular dichroism spectra: inclusion of denatured proteins with native proteins in the analysis. Anal. Biochem. 287, 243–251. 10.1006/abio.2000.4879 11112270

[B35] Sullivan S. P. Koutsonanos D. G. del Martin P. M. Lee J. W. Zarnitsyn V.. (2010). Dissolving polymer microneedle patches for influenza vaccination. Nat. Med. 16, 915–920. 10.1038/nm.2182 20639891 PMC2917494

[B36] Teodorescu M. Bercea M. (2015). Poly(vinylpyrrolidone) - a versatile polymer for biomedical and beyond medical applications. Polym. Plast. Technol. Eng. 54, 923–943. 10.1080/03602559.2014.979506

[B37] van de Weert M. Hennink W. Jiskoot W. (2000). Protein instability in poly(lactic-co-glycolic acid) microparticles. Pharm. Res. 17, 1159–1167. 10.1023/A:1026498209874 11145219

[B38] Veitch N. (2004). Horseradish peroxidase: a modern view of a classic enzyme. Phytochemistry 65, 249–259. 10.1016/j.phytochem.2003.10.022 14751298

[B39] Whitmore L. Wallace B. (2004). DICHROWEB, an online server for protein secondary structure analyses from circular dichroism spectroscopic data. Nucleic Acids Res. 32, W668–W673. 10.1093/nar/gkh371 15215473 PMC441509

[B40] Whitmore L. Wallace B. A. (2008). Protein secondary structure analyses from circular dichroism spectroscopy: methods and reference databases. Biopolymers 89, 392–400. 10.1002/bip.20853 17896349

[B41] Xu B. Jiang G. Yu W. Liu D. Zhang Y. Zhou J. (2017). H_2_O_2_-responsive mesoporous silica nanoparticles integrated with microneedle patches for the glucose-monitored transdermal delivery of insulin. J. Mater. Chem. B 5, 8200–8208. 10.1039/C7TB02082A 32264463

