# Supplementary material for: Fabrication of Circular Obelisk-Type Multilayer Microneedles Using Micro-Milling and Spray Deposition
Source: Front Bioeng Biotechnol. 2018 May 11;6:54. doi: 10.3389/fbioe.2018.00054 (PMC5958193; doi:10.3389/fbioe.2018.00054)
Supplement: Supplementary file 1 [file Data_Sheet_1.docx]

Supplementary Material

Fabrication of circular obelisk-type multilayer microneedles using micro-milling and spray deposition

Min Jung Kim^1^, Seok Chan Park^1^, Binod Rizal^1^, Giselle Guanes^2^, Seung-Ki Baek^3^, Jung-Hwan Park^4^, Amy R. Betz^2^, Seong-O Choi^1^*

^1^Department of Anatomy and Physiology and Nanotechnology Innovation Center of Kansas State, Kansas State University, Manhattan, KS, USA

^2^Department of Mechanical and Nuclear Engineering, Kansas State University, Manhattan, KS, USA

^3^QuadMedicine R&D Centre, QuadMedicine Co., Ltd, Seongnam, Republic of Korea

^4^Department of BioNano Technology and Gachon BioNano Research Institute, Gachon University, Seongnam, Republic of Korea

*** Correspondence:**Seong-O Choi
sochoi@ksu.edu

#
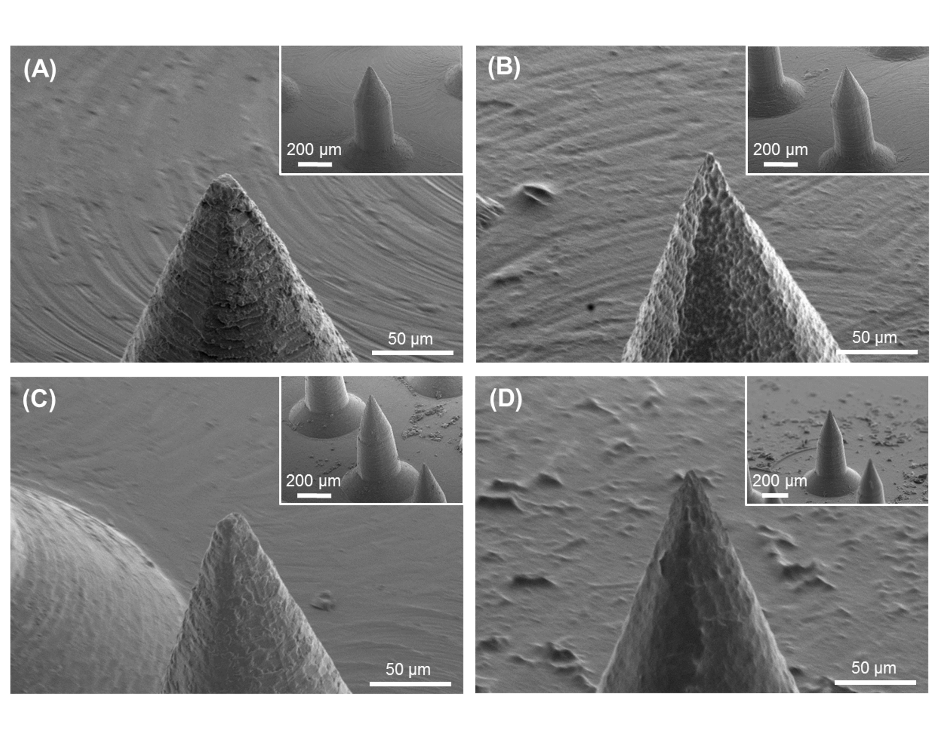
Supplementary Figures

**Supplementary Figure 1.** Effect of oxygen plasma etching on the sharpness of microneedle tips. Tips of circular obelisk needles before (A) and after (B) plasma etching. Tips of beveled-circular obelisk needles before (C) and after (D) plasma etching.


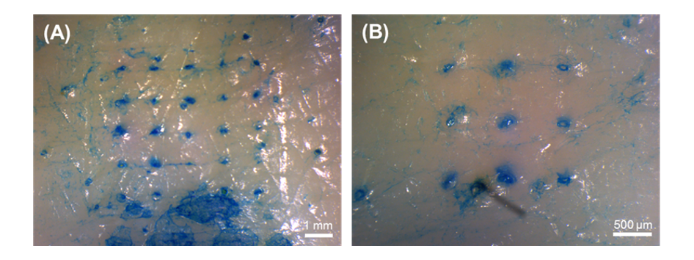
 **Supplementary Figure 2.** Top view of pig cadaver skin after the insertion of (A) Circular obelisk multilayer microneedles and (B) Beveled-circular multilayer microneedles. Insertion sites were stained with a blue tissue marking dye after microneedle removal. Scale bars represent 1 mm and 500 μm for (A) and (B), respectively.

# Supplementary Tables

**Supplementary Table 1.** Mean values and standard deviation of detailed dimensions measured from the aluminum primary masters. (Average ± standard deviation, n=7)

| Microneedle  geometry | Apex angle (degrees, α_apex_) | Bevel angle (degrees, α_bevel_) | Diameter (µm) | | | Height (µm) | | |
| --- | --- | --- | --- | --- | --- | --- | --- | --- |
|  |  |  | D_tip_ | D_stem_ | D_base_ | H_tip_ | H_stem_ | H_base_ |
| Circular obelisk | 57.45 ± 1.31 | 90.24 ± 1.34 | 220.67 ± 6.19 | 220.67 ± 6.19 | 443.16± 4.05 | 210.51 ± 6.11 | 223.99 ± 5.42 | 66.89 ± 4.40 |
| Beveled-circular obelisk | 47.49 ± 0.99 | 84.20 ± 0.11 | 192.24 ± 5.51 | 243.33 ± 1.41 | 451.57 ± 3.73 | 201.18 ± 2.93 | 259.32 ± 3.69 | 89.23 ± 9.21 |
